# Variations in the fecal microbiota and their functions of Thoroughbred, Mongolian, and Hybrid horses

**DOI:** 10.3389/fvets.2022.920080

**Published:** 2022-07-28

**Authors:** Xiaohui Wen, Shengjun Luo, Dianhong Lv, Chunling Jia, Xiurong Zhou, Qi Zhai, Li Xi, Caijuan Yang

**Affiliations:** ^1^Institute of Animal Health, Scientific Observation and Experiment Station of Veterinary Drugs and Diagnostic Techniques of Guangdong Province, Ministry of Agriculture of Rural Affairs, Key Laboratory of Animal Disease Prevention of Guangdong Province, Guangdong Academy of Agricultural Sciences, Guangzhou, China; ^2^Department of Animal Science, College of Biology and Food, Shangqiu Normal University, Shangqiu, China; ^3^National S&T Innovation Center for Modern Agricultural Industry, Guangzhou, China

**Keywords:** 16S rRNA, breed, microbiota, diversity, fibrolytic bacteria

## Abstract

The horse gut is colonized by a rich and complex microbial community that has important roles in horse physiology, metabolism, nutrition, and immune functions. Fewer across-breed variations in horse gut microbial diversity have been illustrated. In this article, the gut microbiota of Thoroughbred, Mongolian, and Hybrid horses [first filial generation (F1) of Mongolian (maternal) and Thoroughbred (paternal)] were studied by second-generation high-throughput sequencing technology. Differences in gut microbiota composition and function between breeds were determined using diversity and functional prediction analysis. The alpha diversity analysis showed that Thoroughbred horses had a more abundant and diverse gut microbiota, while the diversity of gut microbiota in Hybrid horses was intermediate between Thoroughbred and Mongolian horses. Subsequent cluster analysis showed that Hybrid horses have a microbiota composition more similar to Mongolian horses. LEfSe analysis revealed that the bacterial biomarkers for Thoroughbred horses at the family level were Prevotellaceae, Rikenellaceae, Fibrobacteraceae, p_251_o5, Lactobacillaceae, and uncultured_bacterium_o_WCHB1_41; the bacterial biomarker for Mongolian horses was Planococcaceae; and the bacterial biomarkers for Hybrid horses were Moraxellaceae, Enterobacteriaceae, and Ruminococcaceae. The functional prediction results indicated that the metabolic pathways differ significantly between the breeds. Regarding metabolism, the Hybrid horses had the lowest proportion of the carbohydrate metabolic pathways, while the energy metabolic pathway had the highest proportion. The abundance ratios of the remaining eight metabolic pathways in Hybrid horses were between Thoroughbred and Mongolian horses. In conclusion, the results of this study showed an association between horse breeds and gut microbiota.

## Introduction

The horse gut is well developed and colonizes a rich and complex microbiome composed of bacteria, fungi, protozoa, and archaea. The microbial communities interact with the host to maintain gut health. On the one hand, the host gut provides the necessary environmental conditions for microbial growth, such as nutrients, temperature, humidity, and pH (1). On the other hand, the gut microbiota convert complex carbohydrates into short-chain fatty acids (SCFAs) through fermentation, providing the host with essential nutrients and energy (2). Meanwhile, some beneficial microbes in the gut can also specifically bind to the mucosal epithelium to enhance the gut immune protection barrier, thus preventing the invasion of pathogenic microorganisms (3). In addition to nutritional and immune barrier roles, the composition of the gut microbiota and their stability also play an important role in the host behavior (4), metabolism (5), obesity (6), disease (7), and more.

Notably, many studies have found that host age (8), breed, gender, feeding pattern (9), forage type (10, 11), stress (12), and disease (13) in turn affect the composition and function of the gut microbiota. There were some interesting findings on the relationship between horse breeds and gut microbiota. The 16S rRNA analysis of fecal microbiota from Mongolian and Thoroughbred horses living in Inner Mongolia (China) revealed that the relative abundance of 31.25% (5/16) phyla and 40% (30/75) genera was significantly different between the two breeds (14). Another study showed that Thoroughbred horses had higher gut microbiota diversity than Jeju horses in Korea (15). Moreover, the abundance of beneficial commensal bacteria (Lachnospiraceae, *Oscillibacter*, Clostridium_XIVa, etc.) that produce SCFAs to supply the host with more energy sources was also higher in Thoroughbred horses than in Jeju horses (15). When comparing the differences in gut microbiota among six horse breeds in a study by Massacci et al., 27 genera were found significantly different among breeds (16). Approximately 33% of these 27 bacterial genera were reported to be heritable in humans or other animals (17, 18).

In addition to directly studying the effects of host breeds on gut microbiota abundance, some researchers have tried to find causal genes directly associated with gut-specific or core microbiota. Yang et al. (19) verified that the ABO gene is a causal gene affecting the gut abundance difference of Erysipelotrichaceae species and elucidated the mechanism that has important reference significance for cultivating new varieties of grain-saving and fast-growing pigs. Another study reported a strong impact of paternal inheritance on calf hindgut microbiota and growth performance in early life. Further investigation of the correlation between the SNP genotypes and the gut microbiota revealed that the SNP genotypes in the mucin-coding genes were significantly associated with the abundance of the mucin-degrading bacteria in the gut (20). Both studies speculated that host genotypes first affect the colonization of specific bacteria and then shape the gut microbial structure and composition through the interactions between bacteria.

As an important breed in northern China, Mongolian horses have rich genomic diversity (21) and have a variety of excellent traits, such as adaptability, cold resistance, roughage resistance, disease resistance, and good stamina (22, 23). Thoroughbred horses, a horse species bred in 17th-century Britain, were manually selected to meet the desired conditions of speed, temperament, and body size (24). In comparison with Mongolian horses who have substantial stamina (the speed race distance is generally 30–50 km), the suitable race distance of Thoroughbred horses is 1–4 km (25). In addition, due to severe inbreeding, the fecundity of Thoroughbred horses is relatively poor and not resistant to roughage (26, 27). The first filial generation of Mongolian horses (maternal) and Thoroughbred horses (paternal) emerged in the Horqin grassland (Inner Mongolia, China). The hybrid Mongolian-Thoroughbred horses not only inherit the stamina and toughness of Mongolian horses but also combine the size, speed, and good obedience of Thoroughbred horses (28). This study aimed to evaluate the similarities and differences in the composition of gut microbiota and their functions among breeds and to screen the breed-specific bacterial flora. Fecal samples from Thoroughbred, Mongolian, and their derived F1 hybrids under moderately controlled conditions were collected. Subsequently, high-throughput sequencing of the 16S rRNA V3-V4 region of the bacterial DNA was conducted. The differences in the diversity of gut microbiota and their functions across breeds were determined using QIIME 2 and PICRUSt2 software. The results of this study will provide data for the precision feeding of horses.

## Materials and methods

### Sample collection

Thoroughbred horses (4 years old, male, *n* = 5), Mongolian horses (4–6 years old, male, *n* = 5), and Hybrid horses [6 years old, male, F1 hybrid of Mongolian (maternal) and Thoroughbred (paternal) *n* = 5] were provided by Linyi Zoological and Botanical Garden (Linyi, China) for the experiment. The horses were kept under moderately controlled conditions for 4 months before collecting fecal samples and fed hay (oat and alfalfa) and concentrate feeds (Taifeng Animal Husbandry, Shijiazhuang, China), doing moderate exercise every day. The freshly excreted feces were collected in sterilized plastic sealing bags, stored in an icebox, and returned to the laboratory. A portion of each sample (5 g) was placed in a sterilized tube and frozen at −80°C.

### 16S rRNA amplicon sequencing

Fecal microbial genomic DNA was extracted using FastDNA SPIN Soil Kit (MP Biomedicals, Santa Ana, CA). The DNA concentration and purity were determined by UV-vis spectrophotometer UV-1900i (Shimadzu Corporation, Tokyo, Japan), and its integrity was determined by 0.8% agarose gel electrophoresis. The DNA templates extracted above were amplified using universal primers in the V3-V4 region of the bacterial 16S rRNA gene (338F: 5′-ACTCCTAC GGGAGGCAGCA-3′ and 806R: 5′-GGACTACHVGGGTWTCTAAT-3′). The 20 μl reaction system was as follows: DNA 10 ng, 2.5 mmol/L dNTPs 2 μl, 5 × FastPfu buffer 4 μl, BSA 0.2 μl, FastPfu polymerase 0.4 μl, 5 mol/L primers 0.8 μl, adding ddH_2_O supplemented the reaction system. The PCR conditions were 95°C predenaturation for 3 min, 98°C denaturation for 20 s, 58°C annealing for 15 s, and 72°C was extended for 20 s, totaling 30 cycles. Finally, 72°C was maintained for 5 min. The PCR amplification products were detected using a 2% agarose gel electrophoresis and then recovered using a DNA gel extraction kit (Tiangen Biotech, Beijing, China). Sequencing libraries were constructed with the amplified fragments, and then, qualified sequencing libraries were sequenced using the Novaseq 6000 platform (Illumina, CA, USA).

### 16S rRNA gene-based microbiome analyses

The raw data obtained from sequencing were first filtered by Trimmomatic v0.33 software, and then, the primer sequences were removed using cutadapt 1.9.1 software. Subsequently, clean reads of each sample were stitched by overlap using Usearch v10 software, and then, post-splicing length data were filtered based on the length range of different regions. Finally, using the UCHIME v4.2 software, the chimera sequences were removed to obtain effective reads. The operational taxonomic unit (OTU) clustering was performed according to the 97% sequence similarity, using the Usearch v10 software. The number of shared and unique OTU between samples was calculated using the Venn diagram. Characteristic sequences were taxonomic annotated using a naive Bayesian classifier (confidence: 0.7) with SILVA (release 132) as a reference database. Species abundance figures were generated at different taxonomic levels using QIIME 2 software. The rarefaction curves and rank abundance curves were used to evaluate whether the sample sequencing volume met the requirements and the richness and evenness of the sample, respectively.

### Statistical analysis

Alpha diversity was assessed using QIIME 2 software to calculate the Chao 1 index and Shannon index. The Chao 1 index estimates species richness, and the larger the value represents the more species were included in the sample. The Shannon index is used to measure species diversity, influenced by species richness and evenness in the sample microbial community. Differences in alpha diversity indices between groups were analyzed by one-way ANOVA with Tukey's *post-hoc* test, and *P* < 0.05 was considered significant. Beta diversity analysis was processed by QIIME 2 software to compare species diversity between different samples. The unweighted and weighted UniFrac algorithms were used to calculate the distance between samples. UniFrac measures the difference between samples considering phylogenetic linkage. The unweighted algorithm focuses on the existence of a species, while the weighted algorithm takes both existence and abundance into consideration. Linear discriminant analysis (LDA) effect size (LEfSe) was used to search for biomarkers with statistical differences between the groups. First, the OTUs with significant differences in abundance were detected using the nonparametric factorial Kruskal-Wallis sum rank test. Then, LDA was used to estimate the effect of each species on the differential effects. Differences in the microbial community at the genus level were compared using the one-way ANOVA with Tukey's *post-hoc* test, and *P* < 0.05 was considered significant. The correlation network was constructed by performing Spearman's rank correlation analysis and screening for data with a correlation > 0.1 and a *P* < 0.05. PICRUSt2 software was applied to predict potential functional genes based on the Integrated Microbial Genomes database. The difference in functional genes and their effects on KEGG metabolic pathways between groups were analyzed by STAMP software.

## Results

### Sequencing quality assessment

**Figure 1:**
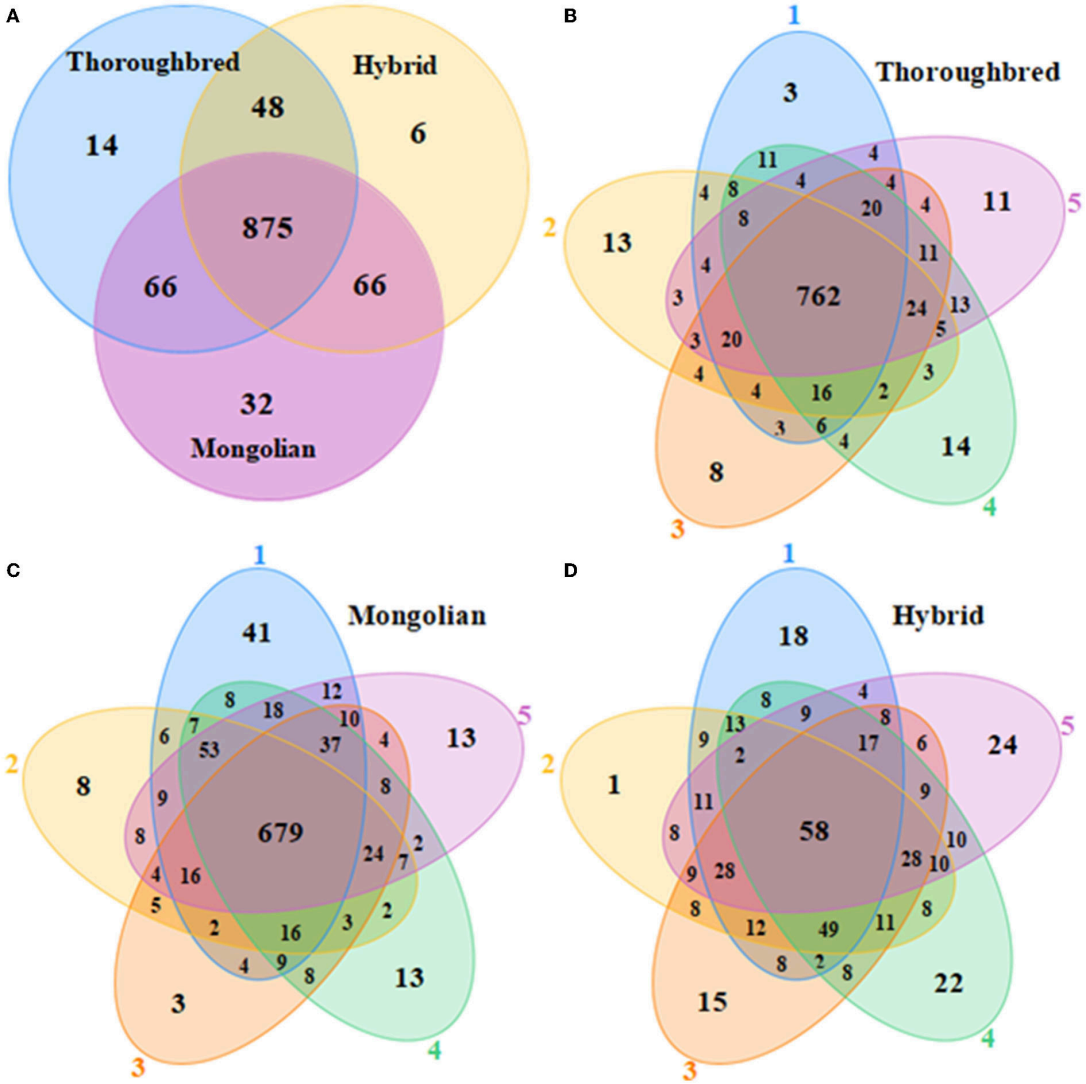
Venn diagrams. **(A)** Venn diagram of OTU distributions between Thoroughbred, Mongolian, and Hybrid horses. **(B–D)** Flower diagrams of OTU distribution between samples in Thoroughbred, Mongolian, and Hybrid horses, respectively.

A total of 1,199,516 pairs of reads were sequenced from 15 samples, and 736,739 effective reads were generated after low quality and length filtering (quality control data are detailed in Supplementary Table 1). In total, 1,107 OTUs were obtained by clustering effective reads at 97.0% similarity levels using the Usearch v10 software and were annotated to 20 phyla and 173 genera. The Venn diagram showed that three groups shared 875 OTUs (Figure 1A). The Thoroughbred horses contained 14 OTUs that were mainly annotated to the phyla Bacteroidetes and Firmicutes. The Mongolian horses contained 32 unique OTUs that were annotated to the phyla Bacteroidetes, Firmicutes, and Spirochaetes. There were six OTUs that belonged exclusively to Hybrid horses, and the mainly annotated phyla were Bacteroidetes and Firmicutes. The number of OTUs shared by each group of samples was 762 (75.97%), 586 (58.89%), and 995 (65.35%), respectively (Figures 1B–D). The rarefaction curve of the samples began to flatten when sampled at around 30 000, indicating sufficient sequencing depth to present most species in the samples (Supplementary Figure 1). The trends of the rank abundance curves were flat, indicating a good evenness of the species composition in the samples (Supplementary Figure 2).

### Effect of breed on gut microbial diversity

**Figure 2:**
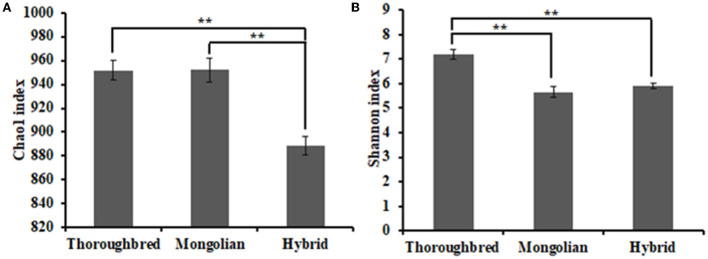
Differential analysis of the alpha diversity index. **(A)** Chao1 index. **(B)** Shannon index. Statistical method: one-way ANOVA with Tukey's *post-hoc* test. **P < 0.01.

**Figure 3:**
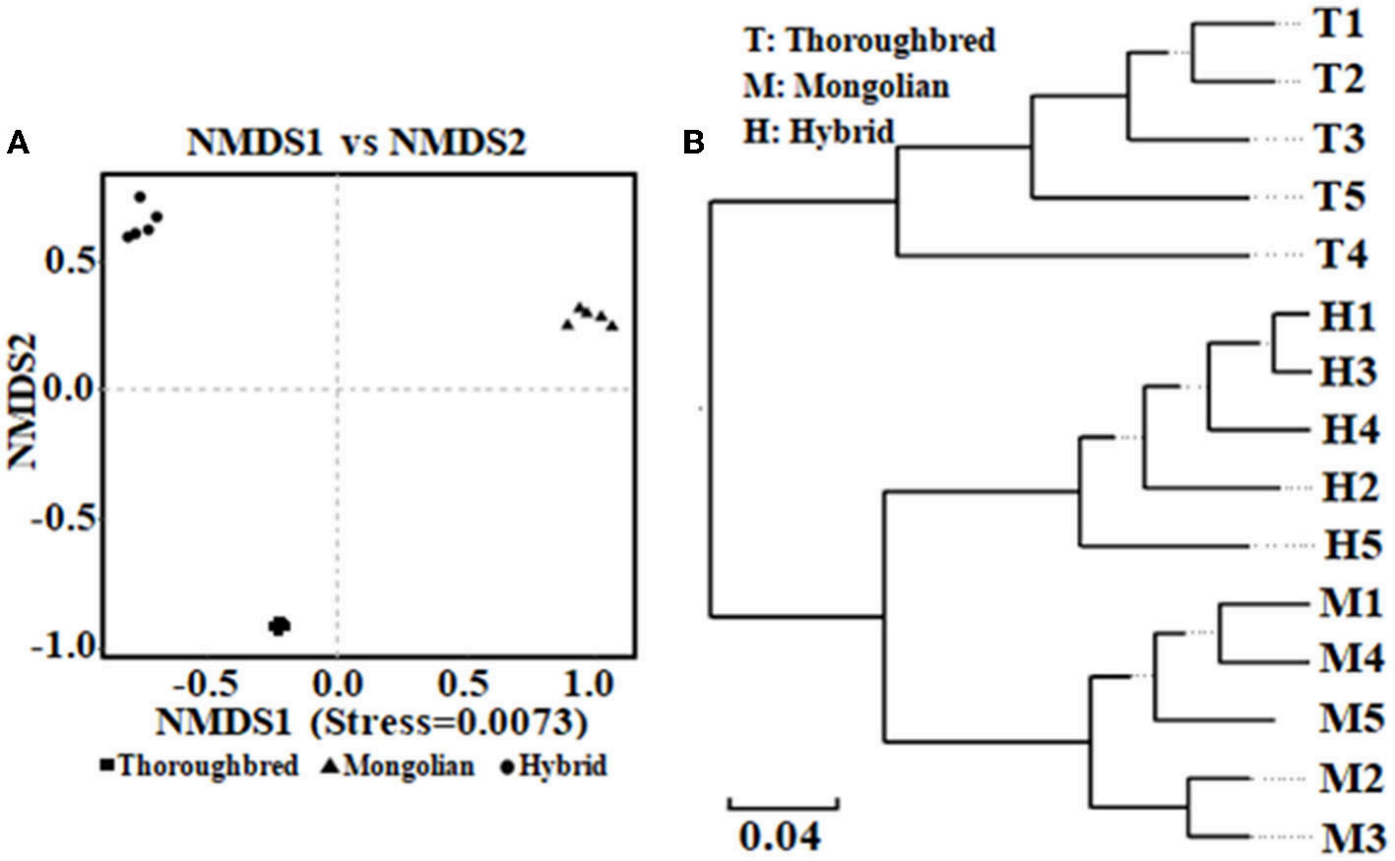
Analysis of beta diversity of microbial community between Thoroughbred, Mongolian, and Hybrid horses. **(A)** NMDS analysis (non-metric multidimensional scaling, using unweighted UniFrac algorithm). **(B)** UPGMA tree (unweighted pair group method with arithmetic mean, using weighted UniFrac algorithm). T, Thoroughbred horses; M, Mongolian horses; H, Hybrid horses.

The Good's coverage was 0.997 8 for Thoroughbred horses, 0.997 5 for Mongolian horses, and 0.996 9 for Hybrid horses, respectively, indicating that the sequencing data were sufficient to cover most species in the samples. The Chao 1 index of Hybrid horses was significantly lower than those in Thoroughbred and Mongolian horses (Figure 2A). The Shannon index of Thoroughbred horses was 7.21, which was significantly higher than that in Mongolian (5.66, *P* < 0.01) and Hybrid horses (5.91, *P* < 0.01) (Figure 2B). The statistical analysis showed that Thoroughbred horses had a more abundant and diverse gut microbiota, while the diversity of gut microbiota in Hybrid horses was intermediate between Thoroughbred and Mongolian horses. The beta diversity analysis was performed based on two distance matrices, namely, non-metric multidimensional scaling (NMDS) and unweighted pair group method with arithmetic mean (UPGMA). The result of NMDS indicated significant differences in gut microbiota among the three groups (Figure 3A). Sample hierarchical clustering trees (Figure 3B) showed that Mongolian and Hybrid horses shared higher similarities in microbial compositions.

### Effect of breed on gut microbial composition

**Figure 4:**
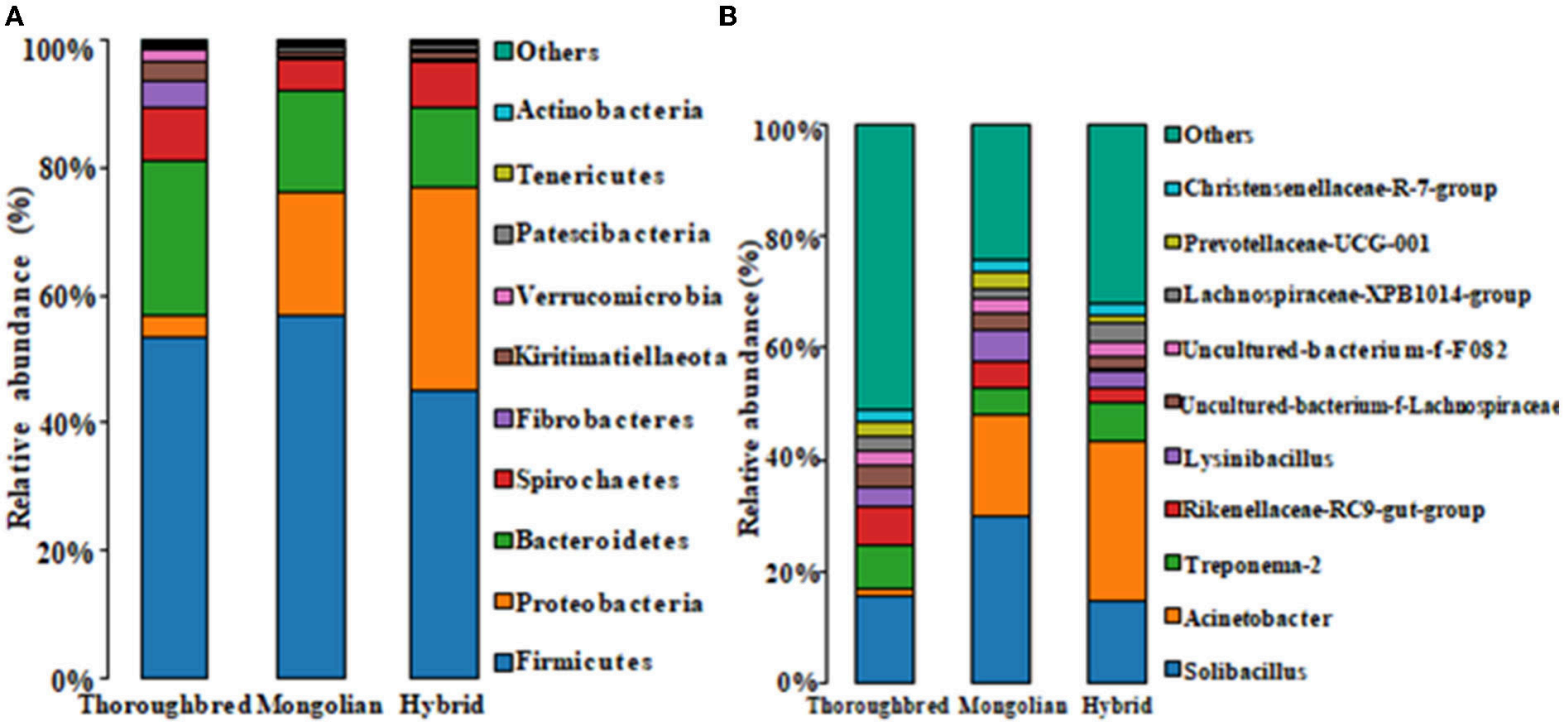
Species distribution histogram. **(A)** The top 10 phyla in abundance. **(B)** The top 10 genera in abundance.

**Figure 5:**
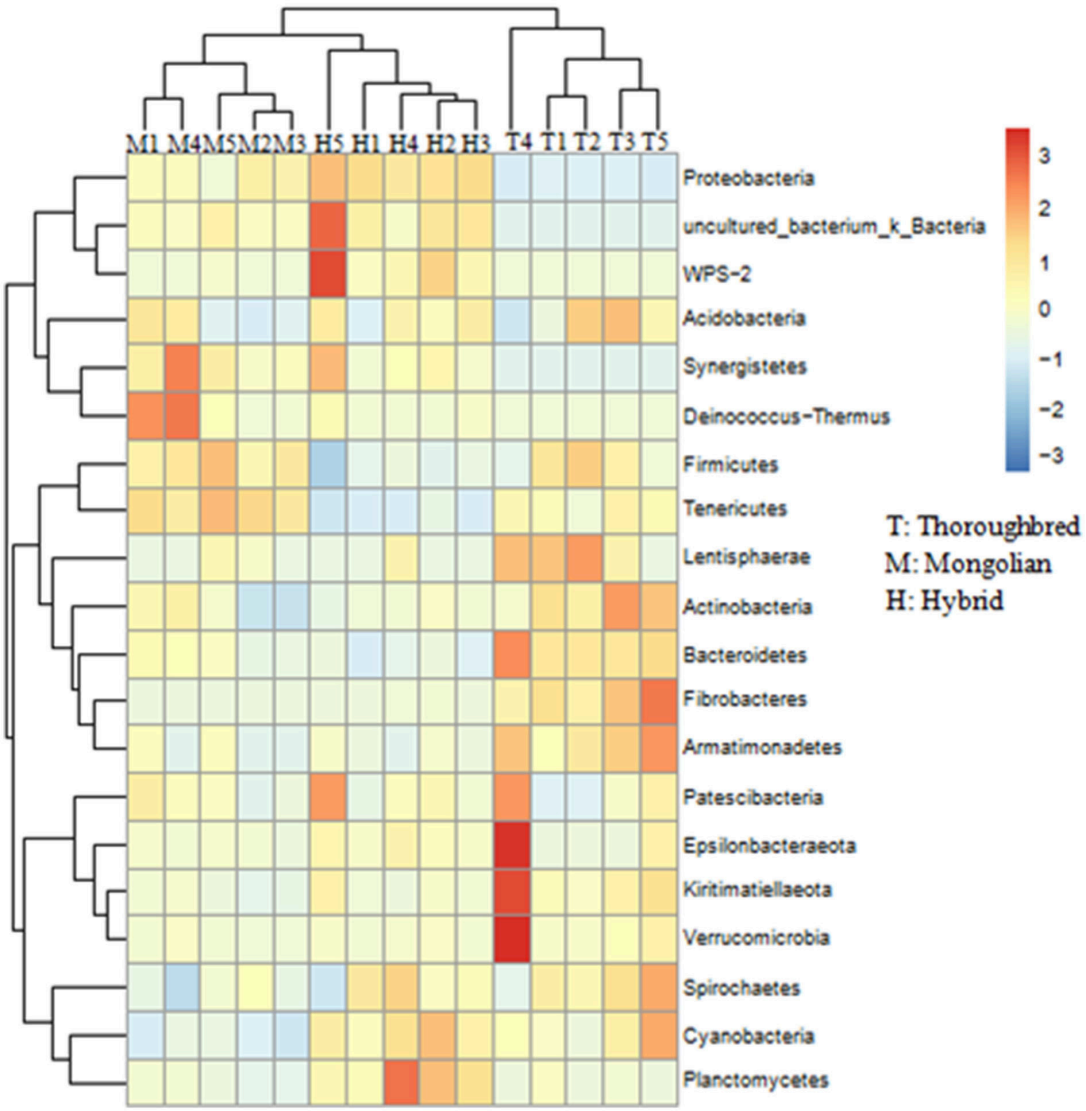
Heatmap of species abundance clustering at the phylum level. T, Thoroughbred horses; M, Mongolian horses; H, Hybrid horses.

The species distribution histogram at the phylum level showed that Firmicutes is the most dominant phylum in the gut of Thoroughbred (53.44%), Mongolian (56.83%), and Hybrid horses (45.02%) (Figure 4A). In addition, among the top 10 phyla in relative abundance, Bacteroidetes, Fibrobacteres, and Actinobacteria were significantly enriched in the gut of Thoroughbred horses. In contrast, the relative abundance of Proteobacteria in Thoroughbred horses was only 3.42%, which is significantly lower than in Mongolian (19.30%) and Hybrid horses (32.04%). Among the top 10 genera, the relative abundances of *Rikenellaceae_RC9_gut_group, uncultured_bacterium_f_Lachnospiraceae*, and *Acinetobacter* were significantly different between the three groups. The relative abundances of *Acinetobacter* and *Lachnospiraceae_XPB1014_group* in the gut of Hybrid horses were significantly higher compared with Thoroughbred and Mongolian horses, while the relative abundances of *Prevotellaceae_UCG-001* and *Rikenellaceae_RC9_gut_ group* were significantly lower (Figure 4B). The heatmap of species abundance cluster at the phylum level showed that the gut microbiota of Mongolian and Hybrid horses were first clustered together and then with Thoroughbred horses, indicating that Mongolian and Hybrid horses have a more similar microbial composition (Figure 5).

**Figure 6:**
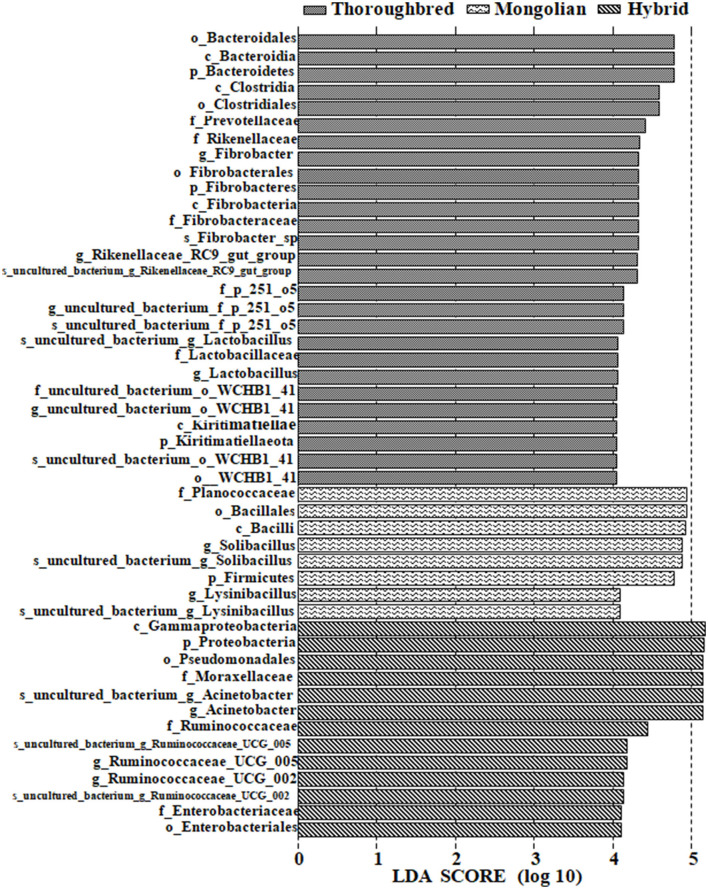
LEfSe [linear discriminant analysis (LDA) effect size] analysis of samples between groups (pairwise Wilcoxon test, LDA scores > 4).

Differences in the abundance of the microbiota between the groups were studied using the one-way ANOVA with Tukey's *post-hoc* test. Among the top 10 phyla, Proteobacteria and Tenericutes varied significantly between the three groups (*P* < 0.01) (Table 1). Genus-level differential analysis revealed that a total of 112 bacterial genera varied significantly between at least two groups (Supplementary Table 2). Compared with the other two groups, the genera not detected in Thoroughbred horses included *Pusillimonas, Mailhella*, and five uncultured bacteria. Mongolian horses lacked *Bifidobacterium, Ruminococcus_2, Shuttleworthia*, and one uncultured bacterium. Meanwhile, Hybrid horses were missing with *dgA-11_gut_group* and one uncultured bacterium. LEfSe analysis showed that Bacteroidales, Clostridiales, Prevotellaceae, Rikenellaceae, *Fibrobacter, Rikenellaceae_RC9_gut_group*, p_251_o5, *Lactobacillus*, WCHB1_41, and Kiritimatiellae were significantly enriched in Thoroughbred horses. The relative abundances of Planococcaceae, Bacillales, *Solibacillus*, Firmicutes, and *Lysinibacillus* were significantly higher in Mongolian horses than in Thoroughbred and Hybrid horses. The relative abundances of Gammaproteobacteria, Pseudomonadales, Enterobacteriaceae, *Ruminococcaceae_UCG_005, Ruminococcaceae_UCG_002*, Moraxellaceae, and *Acinetobacter* were significantly higher in Hybrid horses than in the other two groups (Figure 6).

**Table 1:** Statistical analysis (*P*-value) of the top 10 phyla abundance between paired groups.

**Phylum**	**Thoroughbred vs. Mongolian**	**Thoroughbred vs. Hybrid**	**Mongolian vs. Hybrid**
Proteobacteria	<0.001	<0.001	<0.001
Tenericutes	<0.01	<0.001	<0.001
Bacteroidetes	<0.01	<0.001	≥ 0.1
Fibrobacteres	<0.001	<0.001	≥ 0.1
Actinobacteria	<0.05	<0.05	≥ 0.1
Firmicutes	≥ 0.1	<0.05	<0.01
Kiritimatiellaeota	<0.05	<0.1	≥ 0.1
Verrucomicrobia	≥ 0.1	≥ 0.1	≥ 0.1
Spirochaetes	≥ 0.1	≥ 0.1	≥ 0.1
Patescibacteria	≥ 0.1	≥ 0.1	≥ 0.1

### Correlation network analysis

**Figure 7:**
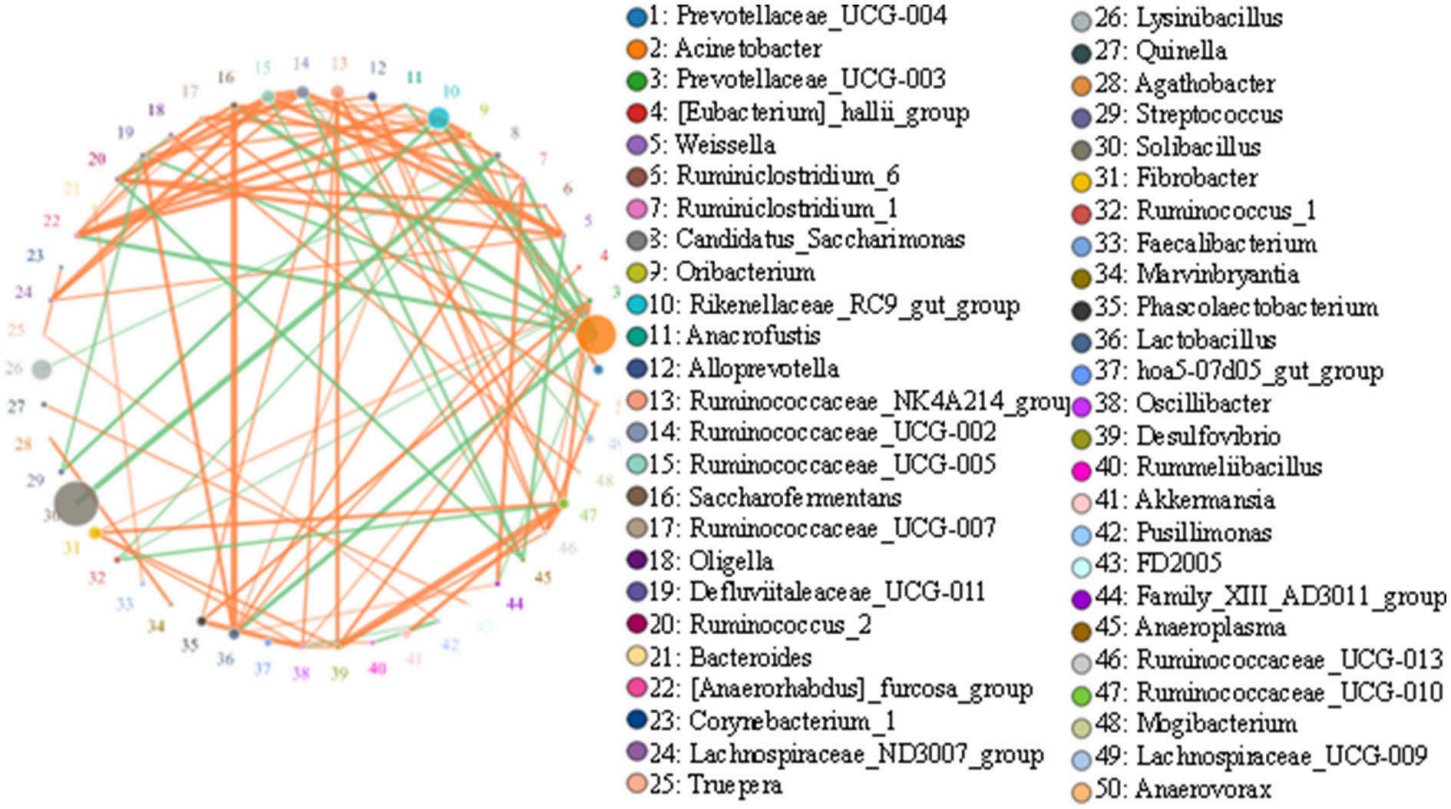
Network diagram of each species at the genus level. Circles represent the species, and circle sizes represent the species' average abundance size. Lines represent the correlation between the two species, and the thickness of the lines represents the strength of the correlation. Orange lines represent a positive correlation, and green lines represent a negative correlation.

Top 50 correlated genera are presented in Figure 7. There was a strong negative correlation between *Solibacillus* and *Candidatus_Saccharimonas* (0.9464). Acinetobacter showed a strong negative association with most genera, such as *Rikenellaceae_RC9_gut_group* (0.9429), *Saccharofermentans* (0.9393), *Lactobacillus* (0.9214), *[Anaerorhabdus]_furcosa_group* (0.9179), *Ruminiclostridium_1* (0.9107), and *Defluviitaleaceae_UCG-011* (0.8893). Rikenellaceae_RC9_gut_group was positively associated with some genera, including *[Anaerorhabdus]_furcosa_group* (0.9214), *Oribacterium* (0.9036), *Ruminiclostridium_1* (0.8964), *Prevotellaceae_UCG-004* (0.8893), *Anaerofustis* (0.8821), *Defluviitaleaceae_UCG-011* (0.875), *Lactobacillus* (0.8536), and *Saccharofermentans* (0.8536), but negatively associated with *Streptococcus* (0.9036) and *Acinetobacter* (0.9429).

### Prediction of 16S rRNA gene function

**Figure 8:**
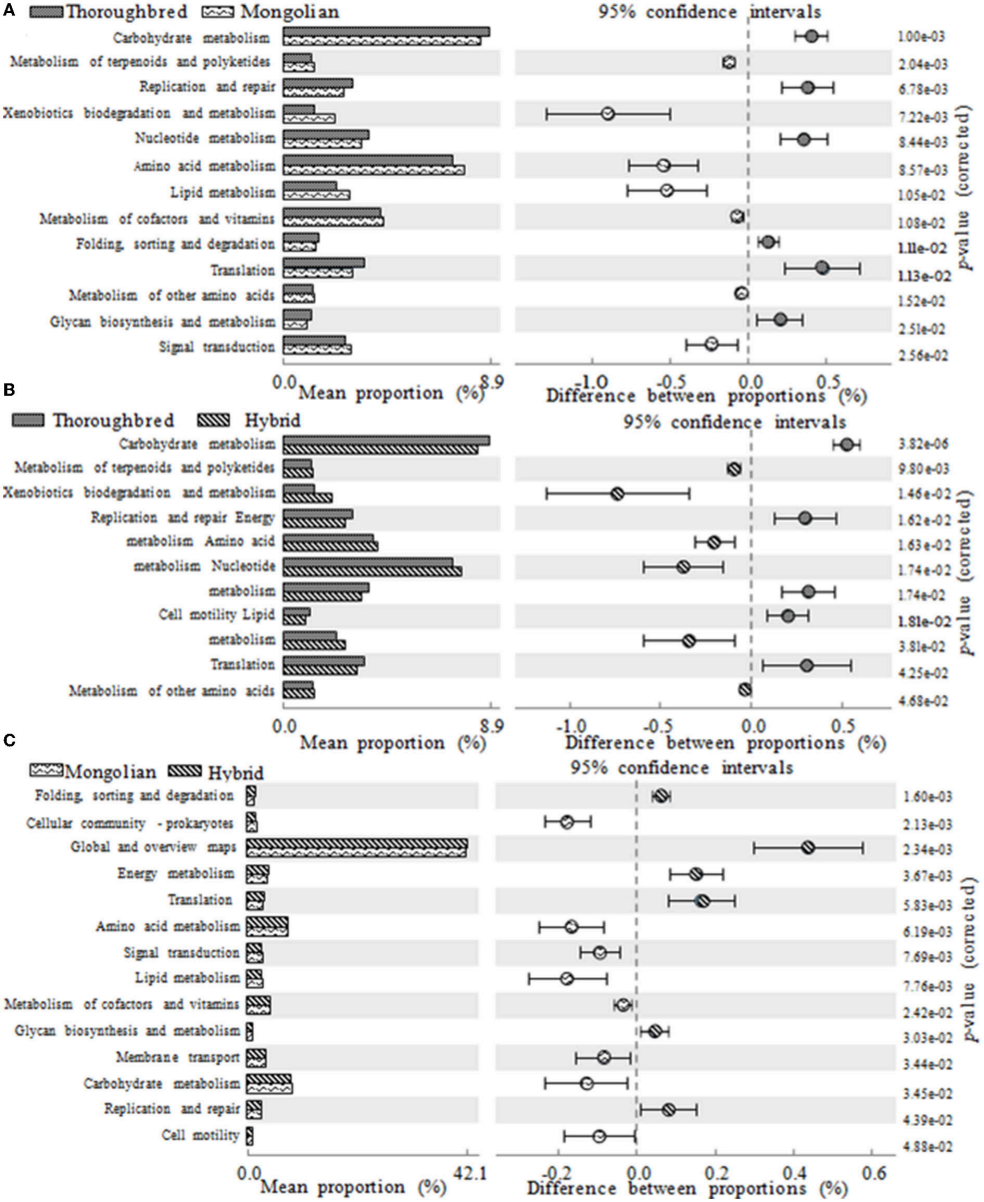
Differences in KEGG (Kyoto Encyclopedia of Genes and Genomes) metabolic pathways between groups (one-way ANOVA with Tukey's *post-test, P* < 0.05). **(A)** The functional gene differences between Thoroughbred and Mongolian horses. **(B)** The functional gene differences between Thoroughbred and Hybrid horses. **(C)** The functional gene differences between Mongolian and Hybrid horses.

The PICRUSt2 prediction results showed that a total of 46 pathways (level 2) were obtained in the 15 fecal samples, mainly involving carbohydrate metabolism (8.55%), amino acid metabolism (7.54%), cofactors and vitamins (4.24%), energy metabolism (3.94%), membrane transport (3.57%), nucleotide metabolism (3.47%), and translation (3.22%). There were 13, 11, and 14 pathways with a relative abundance above 1% that significantly differ between Thoroughbred and Mongolian horses, Thoroughbred and Hybrid horses, and Mongolian and Hybrid horses, respectively. The level two pathways shown in Figure 8 belonged to metabolism, genetic information processing, cellular processes, and environmental information processing. The relative abundance of nucleotide metabolism, translation, and replication and repair pathways was highest in Thoroughbred horses and lowest in Mongolian horses, while in Hybrid horses, it was intermediate. The relative abundance of amino acid metabolism, xenobiotics biodegradation and metabolism, other amino acids metabolism, terpenoids metabolism, and polyketides metabolism was highest in Mongolian horses and lowest in Thoroughbred horses, while in Hybrid horses, it was intermediate. However, the Hybrid horses had the lowest relative abundance of both carbohydrate and lipid metabolism pathways (Figure 8).

## Discussion

Changes in the stability and diversity of gut microbial communities can reflect the gastrointestinal health and growth performance of animals. In this assay, Firmicutes, Bacteroidetes, Spirochaetes, Proteobacteria, Verrucomicrobia, Fibrobacteres, and Kiritimatiellaeota were the predominant phyla, accounting for 95.39, 97.60, and 97.46% of the gut microbiota in Thoroughbred, Mongolian, and Hybrid horses, respectively. Although the predominant phyla identified in this study were the same as the dominant phyla detected by Zhao et al. in studying the gut microbiota difference between Thoroughbred and Mongolian horses, the proportion was different (98% for Thoroughbred horses and 99% for Mongolian horses), and the proportion of each phylum was also different. For instance, the proportion of Bacteroidetes in this study was 24% for Thoroughbred horses and 16% for Mongolian horses, while it was 32 and 33% in Zhao's study; the proportion of Proteobacteria is 3% for Thoroughbred horses and 19% for Mongolian horses in this study, while it was 4 and 1% in Zhao's study (14). There were many reasons for the difference between the two studies, including feeding manners (house feeding vs. grazing), age (4–6 vs. 2–12 years), and gender (male vs. male and female). Notably, the relative abundances of Proteobacteria differ significantly between the three groups in this study, while Kiritimatiellaeota differs significantly only between Thoroughbred and Mongolian horses. The competitive relationship between Firmicutes and Bacteroides has been reported to affect host nutrient uptake and can modulate host obesity genes (29, 30). The Firmicutes/Bacteroides ratio (F/B) of Mongolian and Hybrid horses was significantly higher than that of Thoroughbred horses (3.58, 3.65 vs. 2.20, *P* < 0.01), suggesting that Mongolian and Hybrid horses absorb heat from feed more efficiently than Thoroughbred horses and may be related to cold tolerance properties.

In this study, Prevotellaceae and Rikenellaceae (Bacteroidetes), Fibrobacteraceae (Fibrobacteres), Lactobacillaceae (Firmicutes), WCHB1_41 (Verrucomicrobia), and Kiritimatiellaeota were significantly enriched in the feces of Thoroughbred horses. Prevotellaceae has enzymes and gene clusters capable of fermenting and utilizing complex polysaccharides, and the abundance of the bacterial family in the gut is influenced by breed and gender (31). The RC9 gut group of Rikenellaceae increased the apparent digestibility of acid detergent fiber and neutral detergent fiber, thus improving the dry matter intake of animals (32). Fibrobacteraceae can reduce the inflammatory response and improve production performance by promoting fiber fermentation (33). Some studies have also found a correlation between beef cattle chromosome 27 and Prevotellaceae, Fibrobacteraceae, and RF16 (34). Lactobacillaceae belongs to lactic acid-producing bacteria, but when overgrown, it leads to the occurrence of animal intestinal disease by producing excessive lactate production and reducing the pH of horse hindgut (35). Guo et al. (36) found that Akkermansia and WCHB1_41 were significantly enriched in the gut of yaks during the cold season. Moreover, the functional analysis found that the arginine and fatty acid anabolic pathway encoded by Akkermansia, Kiritimatiellaeota, and WCHB1_41 can effectively improve the efficiency of energy and nitrogen utilization in yaks and facilitate yaks to survive the nutritional stress in the severe cold seasons (36). The above bacteria can produce SCFAs by fermenting the dietary fiber. It is well known that SCFAs can not only provide energy for the intestinal epithelial cells but also firmly maintain the morphology and function of the intestine (37, 38). Moreover, SCFAs have also been confirmed to promote glucose intake and metabolism in skeletal muscle, and butyrate can improve the energy metabolism efficiency of muscle fibers (39, 40). Based on the functions of the above bacterial families (fiber fermentation, inflammation mitigation, stress adaptation, and promotion of SCFAs on energy uptake and metabolism in skeletal muscle), it is speculated that these different bacterial families play a positive role in the performance of Thoroughbred horses.

The biomarkers of Mongolian horses belonged to Firmicutes, including Bacilli, Bacillales, Planococcaceae, and two genera (*Solibacillus* and *Lysinibacillus*). In the study of Xiong et al. (41), gut Bacillales and Planococcaceae were found to be negatively associated with the average daily gain and the final weight of pigs at the end of the trial, but positively associated with the height of small intestinal villi and microvilli (41). *Solibacillus* was found to exist as the predominant bacterial genus in the gut of *Macaca munzala* with metabolic disorders (42). It was found that buffaloes grazing in regions lacking pasture had decreased alpha diversity compared with those grazing in pasture-sufficient regions, and the dominant genus was *Solibacillus*. It was speculated that the enrichment of *Solibacillus* in the buffaloes' gut with decreased food intake is an adaptive response to dietary variability (43). *Solibacillus silvestris* and *Lysinibacillus* sp. D060 have been confirmed to contain many bile acid metabolism genes and bile acid biotransformation ability (44). In addition, the functional prediction results showed that the lipid metabolism of the Mongolian horses was significantly higher than that of the other two groups. Therefore, in combination with the above functions of the biomarkers and the prediction results, it can be speculated that the gut microbiota of Mongolian horses has a positive role in maintaining their roughage resistance and stamina.

The differential species in the gut of Hybrid horses included three families (i.e., Moraxellaceae, Enterobacteriaceae, and Ruminococcaceae) and three genera (i.e., *Acinetobacter, Ruminococcaceae_UCG_005*, and *Ruminococcaceae_UCG_002*). The genus *Acinetobacter* under the family Moraxellaceae contains many opportunistic pathogens and has been reported to cause multiple diseases in horses, such as respiratory tract infections, foal septicemia, foal abortion (45, 46), meningitis (47), endocarditis (48), wound and skin infections (49), and urogenital tract infections (50). Enterobacteriaceae are widely distributed in nature; have a broad host range; and are parasitic or symbiotic in humans, animals, and plants. Some genera in the family Enterobacteriaceae, such as *Escherichia* (51), *Salmonella* (52), *Shigella* (53), and *Klebsiella* (54), belong to the common opportunistic pathogens. Ruminococcaceae are the main microorganisms that convert primary bile acids into secondary bile acids and are also involved in polysaccharide degradation (55). In addition, the Ruminococcaceae in the horse gut have also been proved to have immunomodulatory and anti-inflammatory effects (56). *Ruminococcaceae_UCG-005* showed a significantly positive correlation with eggshell strength and egg weight, presumably achieved by increasing feed conversion in laying hens (57). *Ruminococcaceae_UCG_005* also has a potent cellulolytic capacity (58). *Ruminococcaceae_UCG-002* can degrade various polysaccharides, and its degradation product SCFAs has anti-inflammatory effects (59). Overall, both Mongolian and Hybrid horses are enriched with opportunistic pathogenic bacteria and important polysaccharide digestion bacteria in the gut, compared with Thoroughbred horses.

## Conclusion

To explore the relationship between horse breeds and gut microbiota, 16S rRNA high-throughput sequencing was used to detect the microbiota in horse fecal samples. The comparison of gut microbial diversity among breeds showed that Thoroughbred horses had higher microbial diversity than Mongolian horses, while Hybrid horses were intermediate between them. Cluster analysis indicated that the similarity of the gut microbiota was higher within groups than between groups, and a higher similarity was found between Mongolian and Hybrid horses. The LEfSe analysis showed a greater enrichment of fibrolytic bacteria in the gut of Thoroughbred horses. Meanwhile, the functional prediction results of the gut microbiota also showed a significantly higher abundance of carbohydrate metabolic pathways in Thoroughbred horses than in Mongolian and Hybrid horses. Compared with Thoroughbred horses, the gut of Mongolian horses and Hybrid horses was rich in opportunistic pathogens and beneficial bacteria that can degrade polysaccharides to produce SCFAs. In conclusion, there is an association between horse breed and gut microbiota. The results of this study could lay a theoretical foundation for further understanding and improving the precision feeding system of horses.

## Data availability statement

The datasets presented in this study can be found in online repositories. The names of the repository/repositories and accession number(s) can be found at: https://www.ncbi.nlm.nih.gov/sra/PRJNA812711, PRJNA812711.

## Ethics statement

The animal study was reviewed and approved by Ethics Committee of the Shangqiu Normal University.

## Author contributions

XW conceived and designed the experiments. SL, DL, CJ, and XZ contributed to sample collection and reagents preparation. QZ and CY analyzed the data. XW wrote the manuscript. LX revised the manuscript. All authors reviewed the manuscript.

## Funding

This work was supported by field scientific observation and research of animal diseases in Guangdong Province (Department of Science and Technology of Guangdong Province: 2021B1212050021), Guangdong provincial special fund for modern agriculture industry technology innovation teams (Department of Agriculture and Rural Affairs of Guangdong Province: 2021KJ119), screening and application of anti-foodborne Campylobacter phage (The Education Department of Henan Province: 21B230008), and development and application of feed antibiotic substitute based on immune-antioxidant-gut microecological regulation (Department of Science and Technology of Henan Province: 222102320024).

## Conflict of interest

The authors declare that the research was conducted in the absence of any commercial or financial relationships that could be construed as a potential conflict of interest.

## Publisher's note

All claims expressed in this article are solely those of the authors and do not necessarily represent those of their affiliated organizations, or those of the publisher, the editors and the reviewers. Any product that may be evaluated in this article, or claim that may be made by its manufacturer, is not guaranteed or endorsed by the publisher.
