# Supplementary material for: Variations in the fecal microbiota and their functions of Thoroughbred, Mongolian, and Hybrid horses
Source: Front Vet Sci. 2022 Jul 28;9:920080. doi: 10.3389/fvets.2022.920080 (PMC9366519; doi:10.3389/fvets.2022.920080)
Supplement: Supplementary file 1 [file Table_1.DOCX]

Supplementary Table 1 Quality control results of the sequencing data

| Sample ID | Raw Reads | Clean Reads | Effective Reads | AvgLen  (bp) | Effective  (%) |
| --- | --- | --- | --- | --- | --- |
| Thoroughbred 1 | 80123 | 51046 | 48814 | 422 | 60.92 |
| Thoroughbred 2 | 79816 | 50886 | 48761 | 421 | 61.09 |
| Thoroughbred 3 | 80149 | 51554 | 49644 | 420 | 61.94 |
| Thoroughbred 4 | 80328 | 51083 | 49753 | 419 | 61.94 |
| Thoroughbred 5 | 79748 | 50653 | 49168 | 419 | 61.65 |
| Mongolian 1 | 79689 | 51030 | 49526 | 423 | 62.15 |
| Mongolian 2 | 80134 | 51573 | 49895 | 424 | 62.26 |
| Mongolian 3 | 79705 | 51240 | 49510 | 424 | 62.12 |
| Mongolian 4 | 80230 | 51692 | 49940 | 423 | 62.25 |
| Mongolian 5 | 80275 | 51130 | 49527 | 423 | 61.70 |
| Hybrid 1 | 80003 | 51321 | 49051 | 423 | 61.31 |
| Hybrid 2 | 79575 | 50495 | 48405 | 421 | 60.83 |
| Hybrid 3 | 79694 | 51058 | 48898 | 423 | 61.36 |
| Hybrid 4 | 80028 | 50910 | 48660 | 422 | 60.80 |
| Hybrid 5 | 80019 | 50787 | 47187 | 422 | 58.97 |
