# Supplementary material for: Variations in the fecal microbiota and their functions of Thoroughbred, Mongolian, and Hybrid horses
Source: Front Vet Sci. 2022 Jul 28;9:920080. doi: 10.3389/fvets.2022.920080 (PMC9366519; doi:10.3389/fvets.2022.920080)
Supplement: Supplementary file 2 [file Table_2.DOCX]

Supplementary Table 2 Statistical analysis (*P*-value) of the genus-level microbiota abundance between paired groups

| Genus | Thoroughbred vs Mongolian | Thoroughbred vs Hybrid | Mongolian vs Hybrid |
| --- | --- | --- | --- |
| *Acetitomaculum* | < 0.01 | < 0.01 | ≥0.1 |
| *Acinetobacter* | < 0.001 | < 0.001 | < 0.01 |
| *Actinobacillus* | < 0.01 | < 0.05 | ≥0.1 |
| *Agathobacter* | < 0.001 | < 0.001 | ≥0.1 |
| *Anaerofustis* | < 0.01 | < 0.001 | ≥0.1 |
| *Anaeroplasma* | < 0.01 | ≥0.1 | < 0.001 |
| *Anaerovorax* | < 0.01 | < 0.02 | ≥0.1 |
| *Bacillus* | ≥0.1 | < 0.01 | < 0.02 |
| *Bacteroides* | < 0.001 | ≥0.1 | < 0.001 |
| *Bifidobacterium* | < 0.02 | ≥0.1 | < 0.05 |
| *Butyrivibrio* | < 0.1 | ≥0.1 | < 0.01 |
| *Candidatus_Saccharimonas* | < 0.1 | ≥0.1 | < 0.05 |
| *Candidatus_Soleaferrea* | ≥0.1 | < 0.001 | < 0.001 |
| *Catenisphaera* | < 0.001 | < 0.001 | ≥0.1 |
| *Christensenellaceae_R-7_group* | < 0.1 | ≥0.1 | < 0.01 |
| *Clostridium_sensu_stricto_1* | ≥0.1 | < 0.01 | < 0.001 |
| *Clostridium_sensu_stricto_3* | < 0.05 | < 0.01 | ≥0.1 |
| *Cyanobium_PCC-6307* | < 0.05 | ≥0.1 | < 0.05 |
| *Defluviitaleaceae_UCG-011* | < 0.001 | < 0.001 | < 0.02 |
| *Desulfovibrio* | < 0.001 | < 0.02 | < 0.05 |
| *Enterococcus* | < 0.001 | < 0.01 | < 0.05 |
| *Erysipelatoclostridium* | < 0.02 | < 0.01 | ≥0.1 |
| *Erysipelotrichaceae_UCG-004* | ≥0.1 | < 0.05 | < 0.1 |
| *Escherichia-Shigella* | < 0.01 | ≥0.1 | < 0.001 |
| *FD2005* | ≥0.1 | < 0.02 | < 0.02 |
| *Faecalibacterium* | < 0.01 | ≥0.1 | < 0.02 |
| *Family_XIII_AD3011_group* | < 0.001 | < 0.001 | ≥0.1 |
| *Fibrobacter* | < 0.001 | < 0.001 | ≥0.1 |
| *Klebsiella* | ≥0.1 | < 0.01 | < 0.01 |
| *Lachnospiraceae_AC2044_group* | < 0.05 | < 0.02 | ≥0.1 |
| *Lachnospiraceae_ND3007_group* | ≥0.1 | < 0.01 | < 0.001 |
| *Lachnospiraceae_UCG-002* | < 0.01 | < 0.05 | ≥0.1 |
| *Lachnospiraceae_UCG-006* | ≥0.1 | < 0.02 | < 0.05 |
| *Lachnospiraceae_UCG-009* | < 0.001 | ≥0.1 | < 0.001 |
| *Lachnospiraceae_XPB1014_group* | ≥0.1 | ≥0.1 | < 0.01 |
| *Lactobacillus* | < 0.001 | < 0.001 | ≥0.1 |
| *Lysinibacillus* | < 0.05 | ≥0.1 | < 0.02 |
| *Mailhella* | < 0.01 | ≥0.1 | < 0.05 |
| *Marvinbryantia* | < 0.05 | < 0.05 | ≥0.1 |
| *Mogibacterium* | < 0.02 | ≥0.1 | < 0.01 |
| *Olsenella* | < 0.001 | ≥0.1 | < 0.001 |
| *Oribacterium* | < 0.05 | < 0.001 | ≥0.1 |
| *Oscillibacter* | < 0.001 | < 0.02 | ≥0.1 |
| *Phascolarctobacterium* | < 0.001 | < 0.001 | < 0.01 |
| *Phoenicibacter* | < 0.01 | ≥0.1 | < 0.01 |
| *Prevotellaceae_UCG-001* | ≥0.1 | < 0.02 | < 0.01 |
| *Prevotellaceae_UCG-003* | < 0.001 | < 0.01 | < 0.02 |
| *Prevotellaceae_UCG-004* | ≥0.1 | < 0.001 | < 0.001 |
| *Psychrobacillus* | < 0.1 | < 0.02 | ≥0.1 |
| *Pusillimonas* | < 0.05 | ≥0.1 | < 0.1 |
| *Pygmaiobacter* | < 0.05 | < 0.05 | ≥0.1 |
| *Quinella* | < 0.1 | < 0.01 | ≥0.1 |
| *Rikenellaceae_RC9_gut_group* | < 0.01 | < 0.001 | < 0.01 |
| *Rothia* | < 0.01 | ≥0.1 | < 0.01 |
| *Ruminiclostridium_1* | < 0.1 | < 0.01 | ≥0.1 |
| *Ruminiclostridium_5* | < 0.001 | < 0.001 | ≥0.1 |
| *Ruminiclostridium_6* | ≥0.1 | < 0.01 | < 0.001 |
| *Ruminococcaceae_NK4A214_group* | < 0.01 | < 0.05 | ≥0.1 |
| *Ruminococcaceae_UCG-002* | ≥0.1 | < 0.001 | < 0.001 |
| *Ruminococcaceae_UCG-005* | < 0.001 | < 0.001 | < 0.001 |
| *Ruminococcaceae_UCG-007* | < 0.01 | < 0.01 | ≥0.1 |
| *Ruminococcaceae_UCG-010* | < 0.01 | < 0.05 | ≥0.1 |
| *Ruminococcaceae_UCG-013* | < 0.01 | < 0.01 | ≥0.1 |
| *Ruminococcus_1* | < 0.001 | ≥0.1 | < 0.001 |
| *Ruminococcus_2* | ≥0.1 | < 0.001 | < 0.001 |
| *Rummeliibacillus* | < 0.01 | < 0.02 | ≥0.1 |
| *Saccharofermentans* | < 0.01 | < 0.001 | < 0.02 |
| *Shuttleworthia* | < 0.02 | ≥0.1 | < 0.02 |
| *Solibacillus* | < 0.01 | ≥0.1 | < 0.01 |
| *Streptococcus* | ≥0.1 | < 0.001 | < 0.001 |
| *Terrisporobacter* | ≥0.1 | < 0.02 | < 0.01 |
| *Weissella* | ≥0.1 | < 0.001 | < 0.001 |
| *Z20* | < 0.05 | < 0.05 | ≥0.1 |
| *[Anaerorhabdus]_furcosa_group* | < 0.001 | < 0.001 | < 0.05 |
| *[Eubacterium]_hallii_group* | ≥0.1 | < 0.01 | ≥0.1 |
| *[Eubacterium]_ruminantium_group* | < 0.1 | ≥0.1 | < 0.05 |
| *[Ruminococcus]_torques_group* | < 0.01 | ≥0.1 | < 0.001 |
| *dgA-11_gut_group* | < 0.05 | ≥0.1 | < 0.02 |
| *hoa5-07d05_gut_group* | < 0.01 | ≥0.1 | < 0.1 |
| *uncultured_bacterium_c_Burkholderiales_bacterium_Beta_02* | ≥0.1 | < 0.05 | < 0.05 |
| *uncultured_bacterium_c_MVP-15* | < 0.001 | ≥0.1 | < 0.001 |
| *uncultured_bacterium_c_vadinHA49* | ≥0.1 | < 0.01 | < 0.01 |
| *uncultured_bacterium_f_Acetobacteraceae* | ≥0.1 | ≥0.1 | < 0.02 |
| *uncultured_bacterium_f_Bacteroidales_RF16_group* | < 0.01 | < 0.001 | ≥0.1 |
| *uncultured_bacterium_f_Bacteroidales_UCG-001* | < 0.01 | < 0.01 | ≥0.1 |
| *uncultured_bacterium_f_Burkholderiaceae* | < 0.001 | < 0.05 | ≥0.1 |
| *uncultured_bacterium_f_Clostridiales_vadinBB60_group* | ≥0.1 | < 0.001 | < 0.01 |
| *uncultured_bacterium_f_Coriobacteriales_Incertae_Sedis* | < 0.001 | < 0.01 | ≥0.1 |
| *uncultured_bacterium_f_Eggerthellaceae* | < 0.001 | < 0.001 | ≥0.1 |
| *uncultured_bacterium_f_Enterobacteriaceae* | ≥0.1 | < 0.01 | < 0.02 |
| *uncultured_bacterium_f_Lachnospiraceae* | < 0.05 | < 0.001 | < 0.1 |
| *uncultured_bacterium_f_Marinifilaceae* | ≥0.1 | < 0.001 | < 0.001 |
| *uncultured_bacterium_f_Muribaculaceae* | < 0.001 | < 0.001 | < 0.001 |
| *uncultured_bacterium_f_Peptococcaceae* | ≥0.1 | < 0.001 | < 0.01 |
| *uncultured_bacterium_f_Planococcaceae* | < 0.05 | < 0.05 | ≥0.1 |
| *uncultured_bacterium_f_Prevotellaceae* | < 0.001 | < 0.001 | ≥0.1 |
| *uncultured_bacterium_f_Rikenellaceae* | < 0.1 | ≥0.1 | < 0.01 |
| *uncultured_bacterium_f_Ruminococcaceae* | < 0.001 | < 0.001 | ≥0.1 |
| *uncultured_bacterium_f_Succinivibrionaceae* | ≥0.1 | < 0.001 | < 0.01 |
| *uncultured_bacterium_f_Synergistaceae* | < 0.05 | < 0.1 | ≥0.1 |
| *uncultured_bacterium_f_Syntrophomonadaceae* | < 0.001 | < 0.001 | ≥0.1 |
| *uncultured_bacterium_f_Veillonellaceae* | < 0.001 | < 0.001 | ≥0.1 |
| *uncultured_bacterium_f_p-251-o5* | < 0.001 | < 0.001 | < 0.01 |
| *uncultured_bacterium_k_Bacteria* | ≥0.1 | < 0.01 | ≥0.1 |
| *uncultured_bacterium_o_Absconditabacteriales_SR1* | < 0.001 | ≥0.1 | < 0.001 |
| *uncultured_bacterium_o_Bacteroidales* | < 0.001 | < 0.001 | < 0.1 |
| *uncultured_bacterium_o_Bradymonadales* | ≥0.1 | < 0.001 | < 0.01 |
| *uncultured_bacterium_o_Gastranaerophilales* | < 0.05 | ≥0.1 | < 0.01 |
| *uncultured_bacterium_o_Mollicutes_RF39* | < 0.02 | < 0.001 | < 0.001 |
| *uncultured_bacterium_o_Saccharimonadales* | < 0.001 | < 0.01 | ≥0.1 |
| *uncultured_bacterium_o_WCHB1-41* | < 0.05 | < 0.1 | ≥0.1 |
| *uncultured_bacterium_p_Armatimonadetes* | < 0.001 | < 0.01 | ≥0.1 |
| *Acetitomaculum* | < 0.01 | < 0.01 | ≥0.1 |
| *Acinetobacter* | < 0.001 | < 0.001 | < 0.01 |
| *Actinobacillus* | < 0.01 | < 0.05 | ≥0.1 |
| *Agathobacter* | < 0.001 | < 0.001 | ≥0.1 |
| *Anaerofustis* | < 0.01 | < 0.001 | ≥0.1 |
| *Anaeroplasma* | < 0.01 | ≥0.1 | < 0.001 |
| *Anaerovorax* | < 0.01 | < 0.02 | ≥0.1 |
| *Bacillus* | ≥0.1 | < 0.01 | < 0.02 |
| *Bacteroides* | < 0.001 | ≥0.1 | < 0.001 |
| *Bifidobacterium* | < 0.02 | ≥0.1 | < 0.05 |
| *Butyrivibrio* | < 0.1 | ≥0.1 | < 0.01 |
| *Candidatus_Saccharimonas* | < 0.1 | ≥0.1 | < 0.05 |
| *Candidatus_Soleaferrea* | ≥0.1 | < 0.001 | < 0.001 |
| *Catenisphaera* | < 0.001 | < 0.001 | ≥0.1 |
| *Christensenellaceae_R-7_group* | < 0.1 | ≥0.1 | < 0.01 |
| *Clostridium_sensu_stricto_1* | ≥0.1 | < 0.01 | < 0.001 |
| *Clostridium_sensu_stricto_3* | < 0.05 | < 0.01 | ≥0.1 |
| *Cyanobium_PCC-6307* | < 0.05 | ≥0.1 | < 0.05 |
| *Defluviitaleaceae_UCG-011* | < 0.001 | < 0.001 | < 0.02 |
| *Desulfovibrio* | < 0.001 | < 0.02 | < 0.05 |
| *Enterococcus* | < 0.001 | < 0.01 | < 0.05 |
| *Erysipelatoclostridium* | < 0.02 | < 0.01 | ≥0.1 |
| *Erysipelotrichaceae_UCG-004* | ≥0.1 | < 0.05 | < 0.1 |
| *Escherichia-Shigella* | < 0.01 | ≥0.1 | < 0.001 |
| *FD2005* | ≥0.1 | < 0.02 | < 0.02 |
| *Faecalibacterium* | < 0.01 | ≥0.1 | < 0.02 |
