# Supplementary material for: Variations in the fecal microbiota and their functions of Thoroughbred, Mongolian, and Hybrid horses
Source: Front Vet Sci. 2022 Jul 28;9:920080. doi: 10.3389/fvets.2022.920080 (PMC9366519; doi:10.3389/fvets.2022.920080)
Supplement: Supplementary file 4 [file Table_4.DOCX]

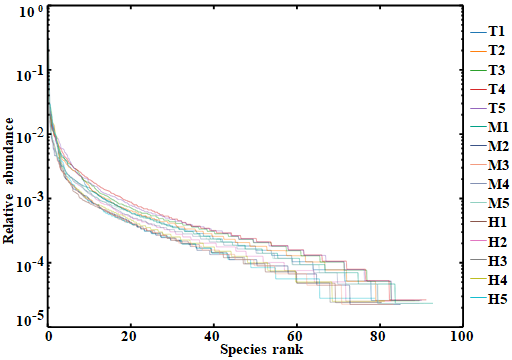


Supplementary Figure 2. Rank abundance curve for 15 samples in all three groups.

T: fecal samples of Thoroughbred horses; M: fecal samples of Mongolian horses; H: fecal samples of Hybrid horses (F1 hybrid: Mongolian (maternal) and Thoroughbred (paternal)).
